# Protein Quality Control Disruption by PKCβII in Heart Failure; Rescue by the Selective PKCβII Inhibitor, βIIV5-3

**DOI:** 10.1371/journal.pone.0033175

**Published:** 2012-03-30

**Authors:** Julio C. B. Ferreira, Berta Napchan Boer, Max Grinberg, Patricia Chakur Brum, Daria Mochly-Rosen

**Affiliations:** 1 Department of Chemical and Systems Biology, Stanford University School of Medicine, Stanford, California, United States of America; 2 School of Physical Education and Sport, University of São Paulo, São Paulo, Brazil; 3 Heart Institute (InCor), University of São Paulo, Medical School, São Paulo, Brazil; Universidade de Sao Paulo, Brazil

## Abstract

Myocardial remodeling and heart failure (HF) are common sequelae of many forms of cardiovascular disease and a leading cause of mortality worldwide. Accumulation of damaged cardiac proteins in heart failure has been described. However, how protein quality control (PQC) is regulated and its contribution to HF development are not known. Here, we describe a novel role for activated protein kinase C isoform βII (PKCβII) in disrupting PQC. We show that active PKCβII directly phosphorylated the proteasome and inhibited proteasomal activity *in vitro* and in cultured neonatal cardiomyocytes. Importantly, inhibition of PKCβII, using a selective PKCβII peptide inhibitor (βIIV5-3), improved proteasomal activity and conferred protection in cultured neonatal cardiomyocytes. We also show that sustained inhibition of PKCβII increased proteasomal activity, decreased accumulation of damaged and misfolded proteins and increased animal survival in two rat models of HF. Interestingly, βIIV5-3-mediated protection was blunted by sustained proteasomal inhibition in HF. Finally, increased cardiac PKCβII activity and accumulation of misfolded proteins associated with decreased proteasomal function were found also in remodeled and failing human hearts, indicating a potential clinical relevance of our findings. Together, our data highlights PKCβII as a novel inhibitor of proteasomal function. PQC disruption by increased PKCβII activity *in vivo* appears to contribute to the pathophysiology of heart failure, suggesting that PKCβII inhibition may benefit patients with heart failure. (218 words)

## Introduction

Maintenance of blood circulation during continual stress, such as hypertension or following cardiac ischemic events and infarction, contributes to cardiac wear and tear and results in accumulation of damaged cardiac proteins leading to cell death and further deterioration of cardiac functions. The cellular protein quality control (PQC) can detect, repair and dispose of cytotoxic damaged proteins using multiple control mechanisms, which include chaperone proteins, the ubiquitin-proteasome system (UPS) and autophagy [1]. The UPS is the primary effector of the PQC process, protecting long-lived cells, such as neurons and cardiomyocytes, from accumulation of aberrant and misfolded proteins [2]. The pathophysiological role of the PQC machinery in the heart emerged from studies showing accumulation of damaged proteins in humans and in animal models with cardiac diseases as well as cardiac mutations in PQC components [3], [4]. There is also up-regulation of proteins involved in UPS and elevated levels of ubiquitinated proteins in hearts of human dilated cardiomyopathy [5]. Some studies found an overall decrease in proteasomal activity associated with and probably contributing to the increased steady state level of ubiquitinated proteins and cell death [5], [6]. However, others reported that several components of the ubiquitin-protein system and/or its overall activity are increased in experimental compensated cardiac hypertrophy and heart failure [7]. Therefore, it remains to be determined whether dysfunction of specific PQC components, such as the UPS, contribute to the development of end-stage heart failure and which signaling events regulate them.

Numerous studies have focused on identifying intracellular nodes where signals converge and serve as multi-effector brakes to suppress or reverse heart failure. We and others have identified PKCβII, which is over activated in failing hearts of humans [8] and in animal models [8], [9], [10], [11], as a potential key player in heart failure. However, the molecular targets of PKCβII are still unknown.

Using human remodeled and failing hearts with different etiologies and two different heart failure models in rats (myocardial infarction-induced and hypertension-induced heart failure; HF), we found a pronounced decline in components of the PQC machinery. Furthermore, we show for the first time that PKCβII, which is over-activated in HF both in humans [8] and in animal models [12], [13], disturbed cardiac PQC by decreasing proteasomal activity. Using different PKC-selective regulators [14], we then demonstrated here that the PKCβII-specific peptide inhibitor, βIIV5-3, prevented the decline in PQC in cultured neonatal cardiac myocytes and that sustained PKCβII inhibition substantially increased survival and cardiac function in myocardial infarction-induced and hypertension-induced heart failure animal models in rats. The molecular bases of these events were also studied.

## Results

### PQC dysfunction parallels heart failure development in an animal model

**Figure 1:**
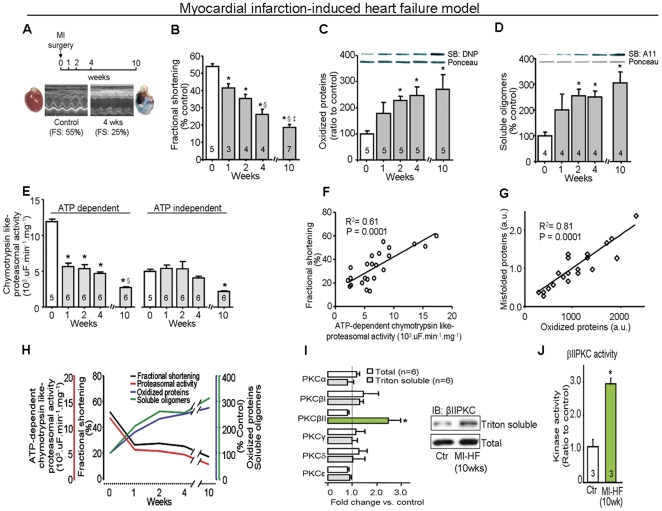
Protein quality control disruption and PKCβII activation during progression of heart failure in a post-myocardial infarction (MI) model of HF in rats. **A**. MI-induced HF protocol (10 weeks follow up) and example of heart morphology and echo data before (left) and 4 weeks after myocardial infarction (right) in rats. **B**. Cardiac fractional shortening, **C**. Oxidized protein levels, **D**. Soluble oligomer level and **E**. Proteasomal activity during the progression of cardiac dysfunction induced by myocardial infarction. *, p<0.05 compared to week 0 (before surgery). §, p<0.05 compared to week 1 after surgery. ‡, p<0.05 compared to week 2 after surgery. **F**. Concordance between fractional shortening and proteasomal activity, and **G**. Concordance between accumulation of misfolded cardiac proteins and oxidized proteins at weeks 0, 1, 2, 4 and 10 after myocardial infarction surgery in rats, **H**. Cardiac fractional shortening, proteasomal activity, oxidized and misfolded protein levels during the progression of cardiac dysfunction induced by myocardial infarction. **I**. The levels of total PKC isozymes (white bars) and translocated active PKCs (gray bars; Triton-soluble proteins or the particulate fraction/total fraction) at 10 weeks after myocardial infarction relative to control, age-matched rats (n = 6). Total proteins and Triton-soluble proteins of the particulate fraction were normalized against GAPDH and Gαo, respectively. Representative blots are of PKCβII total level and translocation to particulate fraction. All biochemical analyses were performed in the ventricular remote area. Error bars indicate s.e.m. *, p<0.05 compared to control-TAT. **J**. PKCβII activity at 10 weeks after myocardial infarction relative to control age-matched rats (n = 3). *, p<0.05 compared to control.

To investigate whether injury-induced progression to heart failure is associated with PQC dysfunction, we evaluated proteasomal activity and accumulation of damaged cardiac proteins in a rat model of myocardial infarction-induced heart failure (Fig. 1A). All measurements were performed in a region remote from the infarcted area in the left ventricle (non-infarcted zone). We found a progressive decline in proteasomal activity during 10 weeks following myocardial infarction that exhibited a tight correlation with the decline in cardiac function (R^2^ = 0.61, p = 0.0001; Fig. 1B, E, F and H), reaching a deficit of 68% and 66%, respectively, when compared with sham-operated rats. The decreased proteasomal activity correlated with an increased accumulation of cardiac oxidized proteins and soluble oligomers of misfolded proteins in the failing hearts (R^2^ = 0.81, p = 0.0001, Fig. 1C, D, G and H). Similar to results observed in human HF hearts [8], [15], we found that, of the PKC isozymes present in rat heart, only PKCβII was activated in the myocardial infarction-induced failed hearts, as evidenced by its increased association with the cell particulate fraction (Fig. 1I); there was also a 3-fold increase in catalytic activity of PKCβII, as compared with that from control rat hearts (Fig. 1J).

### PKCβII activation directly down-regulates proteasomal activity

**Figure 2:**
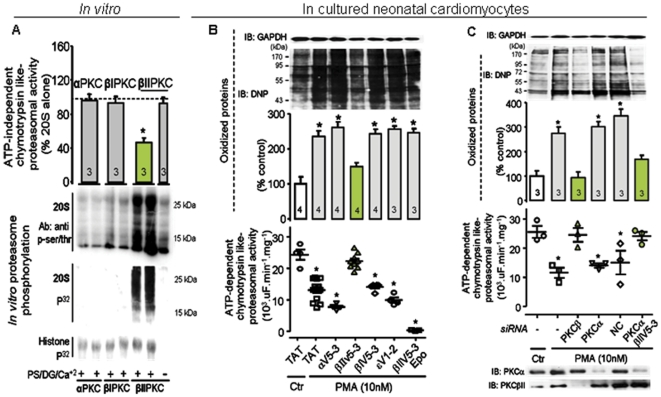
PKCβII activation inhibits proteasomal activity *in vitro* and disrupts protein quality control in cultured neonatal cardiomyocytes. **A**. Proteolytic activity (upper panel) and phosphorylation of purified proteasome 20S (middle and lower panels) by different recombinant PKC isozymes (n = 3 per group). Purified 20S proteasome (1 ug) was individually incubated with recombinant PKCα, PKCβI, PKCβII or PKCε (50 ng) at 37°C for 30 minutes. Proteasome phosphorylation was evaluated using serine/threonine phosphorylation antibody (1∶1000) and [γ^32^P] ATP incorporation. Histone phosphorylation was used to check the effectiveness of different PKC isozymes. Error bars indicate SEM. *, p<0.05 compared to other groups. **B**. Oxidized protein levels (upper panel) and ATP-dependent proteasomal activity (lower panel) in cultured neonatal cardiomyocytes. Cells were stimulated with PMA (a non-specific PKC activator) and the effect of PKC isozyme-specific peptide inhibitors (αV5-3, an αPKC-specific inhibitor; βIV5-3, a βIPKC-specific inhibitor; βIIV5-3, a βIIPKC-specific inhibitor [16]; and εV1-2, an εPKC-specific inhibitor [46]; or epoxomicin were determined. Error bars indicate SEM. *, p<0.05 compared to control-TAT and βIIV5-3-treated groups. Representative blots of oxidized proteins and GAPDH. Oxidized proteins were normalized against GAPDH. **C**. Oxidized protein levels (upper panel) and ATP-dependent proteasomal activity (lower panel) in cultured neonatal cardiomyocytes. Cultured neonatal cardiomyocytes were PKCβ or PKCα down-regulated (siRNA) plus/minus βIIV5-3 and challenged with phorbol ester (PMA). NC, negative control for siRNA. Representative blots of PKCβII and PKCα showed siRNA effectiveness. Oxidized proteins were normalized against GAPDH. Error bars indicate SEM. *, p<0.05 compared to control (non-treated cells), PKCβ down-regulated treated cells and PKCα down-regulated treated cells plus βIIV5-3.

We next determined whether PKCβII directly regulates proteasomal activity. Active PKCβII (but not αPKC or PKCβI) phosphorylated and decreased the proteolytic function of the purified 20S proteasome by 55%, *in vitro* (Fig. 2A, bottom and top panel, respectively). PKCβII activation-mediated proteasomal phosphorylation was essential to decreased proteasomal activity, since inactive PKCβII (added in the absence of its activators, PS/DG/Ca^+2^) had no effect on proteasomal function (Fig. 2A). Further, treatment of cultured neonatal cardiomyocytes with 10 nM phorbol ester 12-myristate 13-acetate (PMA, an activator of most PKC isozymes) for 30 min induced oxidized protein accumulation, at least in part, by decreasing the 26S proteasomal ATP-dependent proteolytic activity (40% decrease) (Fig. 2B). Using selective peptide inhibitors for PKC isozymes, we found that only βIIV5-3, a PKCβII-specific peptide inhibitor [14], [16], [17], [and not selective inhibitors for α, βI or PKCε (αV5-3 and βIV5-3 and εV1-2, respectively), which are also present in cardiac myocytes; [18], [19]] prevented this PMA-induced proteasomal inhibition and increased accumulation of damaged proteins (Fig. 2B). Confirming the pharmacological effect of βIIV5-3, PKCβ knockdown using siRNA similarly abrogated PMA-induced proteasomal inhibition and oxidized protein accumulation in neonatal cardiomyocytes (Fig. 2C). In contrast, PKCα knockdown did not restore PMA-induced proteasomal activity unless cells were pre-treated also with βIIV5-3 peptide (Fig. 2C). In addition, βIIV5-3 treatment prevented damaged protein accumulation and diminished cell death in hydrogen peroxide-treated neonatal cardiomyocytes (Fig. S1) and epoxomicin (a selective proteasome inhibitor) abrogated the cytoprotective effect of βIIV5-3.

### Sustained PKCβII inhibition and cardiac PQC in the myocardial infarction-induced heart failure model

**Figure 3:**
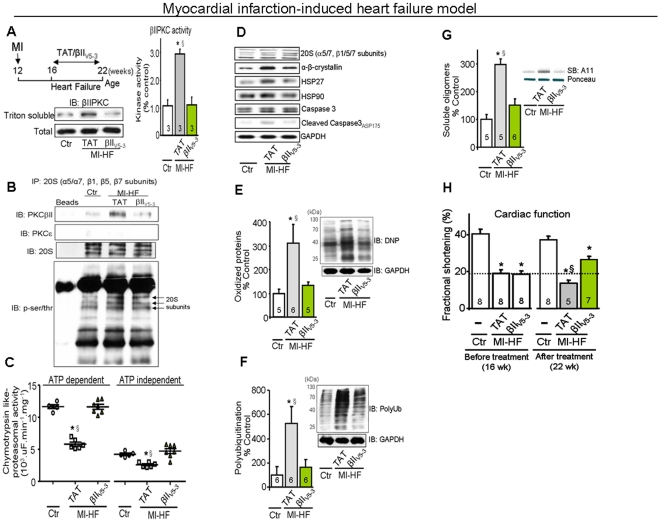
Sustained PKCβII inhibition re-establishes protein quality control and improves cardiac function in myocardial infarction-induced model of heart failure. **A**. Schematic panel of PKCβII treatment in the post-MI heart failure model, representative blots of PKCβII total level and translocation to particulate fraction, and PKCβII activity from left ventricle tissue from 22-week-old myocardial infarction-induced heart failure (10 wks after MI surgery) TAT-treated, βIIV5-3-treated and control (sham) rats (n = 3 per group). **B**. 20S proteasome subunits (α5/7, β1, β5 and β7) were precipitated from left ventricle tissue from 22-week-old myocardial infarction-induced heart failure (10 wks after MI surgery) TAT-treated, βIIV5-3-treated and control (sham) rats (n = 3 per group), and then probed with PKCβII, PKCε and anti-serine and threonine phosphorylation antibodies. Equal sample loading was verified using α5/7, β1, β5 and β7 proteasome subunits antibody. **C**. ATP-dependent and -independent cardiac proteasomal activity. **D**. Representative blots of proteasome 20S, α-β-crystallin, HSP27, caspase-3, cleaved caspase-3 and GAPDH in heart samples from 22 week-old rats (10 wks after MI surgery) (n = 6 per group). Data quantification and statistical details are in supplementary Fig. 4. **E**. Oxidized protein levels, **F**. polyubiquitinated protein levels and **G**. soluble oligomer accumulation in heart samples from control (sham, white bars), TAT-treated (gray bars) and βIIV5-3-treated (green bars) heart failure rats as determined by Western blot (E, F) and slot-blot analysis (G). **H**. Average fractional shortening data from each group at 16 weeks and 22 weeks. All biochemical analyses were performed in the ventricular remote area. Error bars indicate SEM. *, p<0.05 compared to control (sham) rats. §, p<0.05 compared to βIIV5-3-treated heart failure rats.

To evaluate the effect of PKCβII on cardiac PQC in heart failure, we further determined whether sustained administration of βIIV5-3 in a myocardial infarction-induced heart failure model in rats (Fig. 3A) affected cardiac PQC, cardiac function and survival. PKCβII (but not PKCε, an abundant isozyme in the heart) co-immunoprecipitated with the proteasome and decreased its activity in these failing hearts (Fig. 3B and C). After the establishment of HF (4 weeks after myocardial infarction; MI), a subsequent six-week treatment with βIIV5-3 abolished the increased cardiac PKCβII translocation and activity (Fig. 3A), but not the activity of α, βI, δ, γ and PKCε (Fig. S2), and diminished the co-immunoprecipitation of PKCβII and the 20S proteasome as well as its phosphorylation (Fig. 3B). This βIIV5-3 treatment resulted also in a two-fold increase in both ATP-dependent (26S) and -independent (20S) cardiac proteasomal activity back to control levels (Fig. 3C). There were no changes in the protein levels of cardiac proteasome subunits in failing hearts regardless of the treatment (Fig. 3D and Fig. S3). However, sustained PKCβII inhibition completely suppressed the accumulation of cardiac oxidized proteins, polyubiquitinated proteins and soluble oligomers of misfolded proteins in these rat samples (Fig. 3E–G). The increased abnormal protein accumulation in failed non-treated hearts was accompanied by an ∼50% increase in the levels of the small chaperones, α-β-crystallin and HSP27, and a two-fold increase in caspase 3 activation, effects that were reversed by the sustained PKCβII inhibition (Fig. 3D and Fig. S3). In addition, chronic PKCβII inhibition abolished the HF-induced increase in the levels of well-known proteasome substrates, IkB and p53 (Fig. S3).

**Figure 4:**
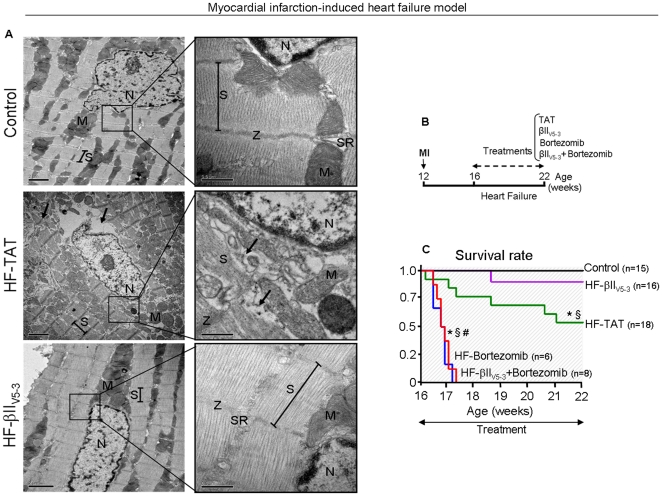
Protein quality control re-establishment by PKCβII inhibition improves survival and cardiac integrity in myocardial infarction-induced model of heart failure. **A**. βIIV5-3 improved cardiac integrity in the post-MI-induced HF model in rats. Heart samples were collected from 22 week-old control (sham), TAT-treated and βIIV5-3-treated heart failure rats (10 wks after MI surgery). Samples from a non-infarcted area (contractile zone) were processed for electron microscopy analysis. The integrity of cardiac myofibril structure (arrow head) was analyzed in each group (n = 3 per group). N, nucleus; S, sarcomere; SR, sarcoplasmic reticulum; Z, Z disc; M, mitochondria. **B**. Schematic panel of sustained treatment with TAT, βIIV5-3, bortezomib or bortezomib plus βIIV5-3 in post-myocardial infarction-induced heart failure in rats. **C**. βIIV5-3 improved survival of rats with post-myocardial infarction-induced heart failure. Note that sustained treatment of heart failure rats with a specific proteasome inhibitor (bortezomib, 0,2 mg/kg, thrice weekly) either alone or combined with βIIV5-3 culminated in 100% mortality as well as abrogated the cardioprotective effects of βIIV5-3 treatment. Control (sham) rats treated with bortezomib did not present increased mortality. Error bars indicate SEM. *, p<0.05 compared to control (sham) rats. §, p<0.05 compared to βIIV5-3-treated heart failure rats. #, p<0.05 compared to TAT-treated heart failure rats.

Importantly, a six-week treatment with βIIV5-3 of animals with HF resulted in a significant increase of fractional shortening compared to non-treated heart failure animals (27±2 *vs.* 14±2%, respectively; Fig. 3H). βIIV5-3 treatment also decreased left ventricular end-diastolic diameter compared to non-treated heart failure animals (8.5±0.3 *vs.* 10.2±0.2 mm, respectively). The improved cardiac function following βIIV5-3 treatment (from 19% before treatment to 27% after 6 weeks of βIIV5-3 treatment) was likely due to improved integrity of cardiac myofibril structure in the treated hearts, as evidenced by electron microscopy (Fig. 4A). Improved integrity of cellular structures was also found in βIIV5-3-treated rats relative to non-treated heart failure animals (Fig. 4A arrows). Finally, PKCβII inhibition strikingly improved HF animal survival, whereas sustained bortezomib treatment (a proteasome inhibitor) abrogated βIIV5-3 therapy-mediated survival improvement in HF rats and accelerated death in rats with HF (Fig. 4B–C), but not in control rats (none of the control animals died after chronic treatment with bortezomib). Further, bortezomib-treated HF rats did not present any sign of drug toxicity, based on anatomopathological analysis. The increased mortality rate of rats with HF in the presence of a proteasome inhibitor, together with our *in vitro* and cell culture experiments, further support our hypothesis that PKCβII inhibition-associated improvements in heart function/survival is mediated, at least in part, by better proteasomal function.

### Sustained PKCβII inhibition and cardiac PQC in the hypertension-induced heart failure model

**Figure 5:**
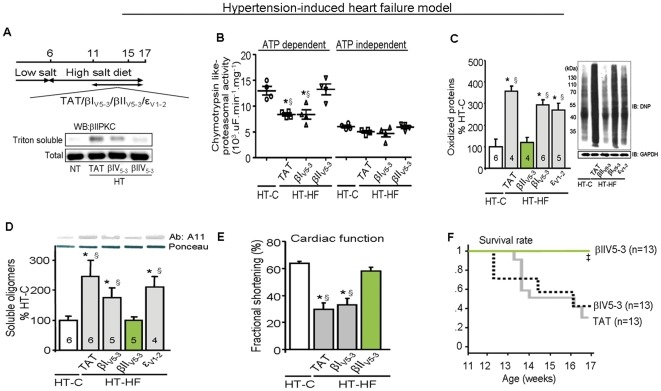
PKCβII inhibition repairs protein quality control in hypertension-induced heart failure model in rats. **A**. Schematic panel of sustained PKCβI, βII or ε inhibition in hypertension-induced model of heart failure in rats. Representative blots of PKCβII total level and translocation to particulate fraction. **B**. ATP-dependent and -independent cardiac proteasomal activity, **C**. Oxidized proteins (as determined by Western blot) and **D**. cardiac soluble oligomer accumulation (as determined by slot blot) in heart samples from 17 week-old normotensive rats (white bar), TAT-treated (gray bar), βIV5-3-treated (gray bar), βIIV5-3-treated (green bar) and εV1-2-treated (gray bar) hypertensive rats. **E**. Average fractional shortening data from each group at the age of 17 weeks-old. **F**. βIIV5-3 improved survival of rats with hypertension-induced heart failure. Error bars indicate SEM. *, p<0.05 compared to control (sham) rats. §, p<0.05 compared to βIIV5-3-treated heart failure rats. Data from Fig. b–d were analyzed by one-way analysis of variance (ANOVA) with *post-hoc* testing by Tukey. Survival was analyzed by the standard Kaplan-Meier analysis with log-rank test.

We next determined whether the role of PKCβII and PQC in heart failure is independent of the etiology of HF, using a hypertension-induced heart failure model in Dahl salt-sensitive rats (Fig. 5A). When placed on a high-salt diet from the age of 6 weeks, Dahl rats exhibit high blood pressure (230 *vs.* 160 mmHg), as previously reported [13]. These hypertensive rats develop compensatory left ventricular hypertrophy by the age of 11 weeks, and die from heart failure between 16 to 21 weeks [13], [20]. Here we show that 17 week-old hypertensive Dahl salt-sensitive rats also exhibit decreased cardiac proteasomal ATP-dependent proteolytic activity and increased levels of oxidized proteins and soluble misfolded protein oligomers (Fig. 5B–D). Similar to the effect of PKCβII inhibition in the myocardial infarction-induced HF model in rats, sustained treatment with βIIV5-3 (but not the βI inhibitor) between weeks 11–17, reduced PKCβII activity to basal levels (Fig. 5A), restored ATP-dependent proteasomal activity and decreased the levels of misfolded cardiac proteins to those seen in control animals (Fig. 5B–D). Importantly, βIIV5-3 (but not the βI inhibitor) treatment prevented the decrease in fractional shortening in hypertensive rats (Fig. 5E). Further, while treated with the PKCβII inhibitor (between weeks 11–17), none of the hypertensive rats died, as compared with 60% death of hypertensive rats treated with vehicle control or the selective inhibitor of the alternatively spliced form, βIPKC (Fig. 5F).

### PQC in left ventricular remodeling and heart failure in humans

**Figure 6:**
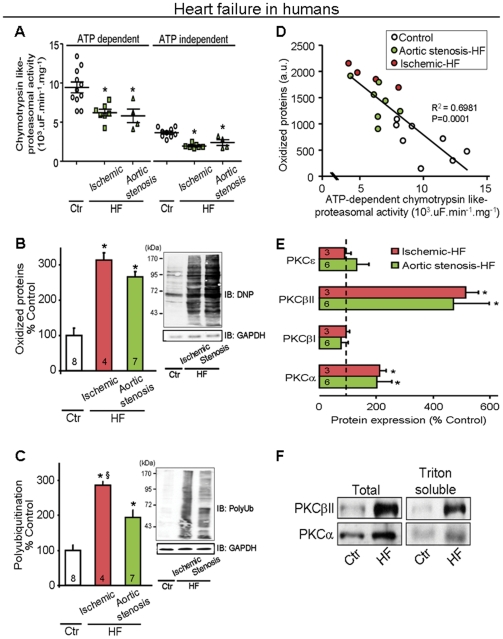
Impaired protein quality control in left ventricular remodeling and heart failure in humans. **A**. ATP-dependent and -independent proteasomal activity, **B**. oxidized protein levels (determined by Western blot) and **C**. polyubiquitinated protein levels (determined by Western blot) in biopsied hearts from aortic stenosis-induced left ventricular remodeling (LVR) patients (green bars), ischemic cardiomyopathy-induced heart failure patients (red bars) and autopsied non-failing human hearts (white bars). **D**. Negative correlation between proteasomal function and oxidized protein accumulation in failing (aortic stenosis-LVR and ischemic-HF) and non-failing heart samples. **E**. Total PKC levels in failing hearts compared to non-failing hearts and **F**. Representative blots of PKCβII and PKCα proteins in total and Triton-soluble fraction (particulate fraction) in biopsied hearts from aortic stenosis-induced left ventricular remodeling patients (n = 6, green bars) and ischemic cardiomyopathy-induced heart failure patients (n = 3, red bars) compared to autopsied non-failing human hearts (n = 6, trace). Total and Triton-soluble fractions were normalized against GAPDH and Gαo, respectively. Error bars indicate SEM. *, p<0.05 compared to control (non-failing heart). §, p<0.05 compared to aortic stenosis-LVR patients.

To determine the extent of PQC disruption in cardiac remodeling/failure, we used heart biopsies from seven patients with aortic stenosis-induced left ventricular remodeling who underwent aortic valve replacement. Heart biopsies from four patients with ischemic cardiomyopathy-induced HF and from autopsy specimens of 13 non-failing human hearts as controls were also examined (Table S1). Despite preserved systolic function, all patients with aortic stenosis displayed heart failure signs and symptoms, presenting functional class III–IV of the New York Heart Association [21]. Both ATP-dependent (26S) and -independent (20S) proteasomal activities were lower by about 50% in aortic stenosis and ischemic failing hearts as compared with controls (Fig. 6A) and the levels of oxidized and polyubiquitinated cardiac proteins were two to three-fold higher in these patients as compared with control subjects (Fig. 6B–C). There was a negative correlation between proteasomal function and oxidized cardiac protein accumulation in failing human hearts (R^2^ = 0.70, p = 0.001; Fig. 6D). There is an obvious caveat of using autopsied hearts as a control. Nevertheless, when we examined PQC, an ATP-dependent function, we found a better PQC in the autopsied samples of control subjects relative to the biopsy samples from remodeled and failing human hearts, suggesting an impaired PQC in failing human hearts.

Because protein kinase C (PKC) isozymes have been implicated in HF [11], [22] we determined whether specific PKC isozymes play a role in proteasomal activity and PQC in this disease. Similar to previous reports [15], [23], both aortic stenosis and ischemic failing human hearts had a five-fold increase in total PKCβII levels, a two-fold increase in PKCα and no changes in the levels of ε and PKCβI relative to controls (Fig. 6E). Elevated PKCβII and PKCα protein levels were accompanied by their activation in failing hearts, as evidenced by their increased association with the cell particulate fraction (Fig. 6F).

## Discussion

**Figure 7:**
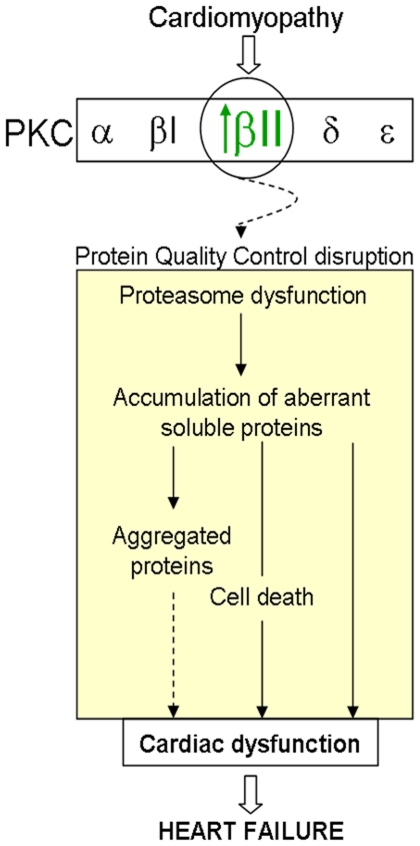
Scheme depicting a possible mechanism of PKCβII-mediated PQC disruption during heart failure establishment.

In the present study, we showed that human hypertrophied and failing hearts of different etiologies, as well as myocardial infarction- and hypertensive-induced heart failure rat models displayed UPS dysfunction-mediated PQC disruption and elevated PKCβII protein activity. We also demonstrated for the first time that PKCβII activation resulted in decreased proteasomal activity and accumulated damaged proteins. Moreover, improved proteasomal function by sustained inhibition of PKCβII, using the highly selective PKCβII inhibitor peptide, βIIV5-3 [16], significantly improved cardiac PQC, ventricular function and survival of myocardial infarction- and hypertensive-induced heart failure models in rats. Of interest, sustained proteasomal inhibition (by bortezomib treatment) abrogated PKCβII-mediated cardioprotective effects and resulted in elevated mortality in the myocardial infarction-induced rat heart failure model. Thus, PKCβII hyper-activation appears to contribute to UPS dysfunction-mediated PQC disruption and subsequent decreased cardiomyocyte viability, cardiac function and survival in HF (Fig. 7).

UPS-mediated PQC disruption has been involved in several chronic degenerative diseases, including neurodegenerative diseases, cancer and cardiac ischemia [24], [25], [26], where UPS malfunction culminates in accumulation of abnormal protein-mediated cellular dysfunction and apoptosis. These findings are extended to human heart failure, since we demonstrate here a ∼50% decrease in proteasomal activity and an ∼3-fold increase in both polyubiquitinated and oxidized proteins in human failing hearts. Furthermore, decreased proteasomal activity was significantly correlated with increased accumulation of oxidized proteins in the failing human hearts (R^2^ = 0.70, p = 0.0001, Fig. 6D). Thus, cardiac dysfunction, decreased UPS activity and PQC inadequacy seem to be common phenomena in developing heart failure, and the progressive PQC disruption paralleled cardiac function decline after myocardial infarction in rats (Fig. 1). Although the data using human-derived tissue samples were similar to those we obtained from the two animal models of HF, there is an inherent variability in disease type and comorbidity-associated factors. Further caveat is the use of pathological specimens from subjects who died of causes other than HF; the cause of death and the timing of sample collection relative to the time of death may introduce additional variable and therefore are not optimal controls. Despite these limitations, the similar findings in humans, in two animal models and in culture support our conclusion regarding the role of PKCβII in regulation of proteasomal function in failing hearts.

A number of studies have shown that activation of PKC contributes to a variety of heart diseases by targeting contractile myofilaments, mitochondrial proteins and transcriptional factors [26], [27]. Different PKC isozymes have been implicated in HF [10]. Similar to previous findings from failed human hearts [15], [23], we found that both aortic stenosis and ischemic-failing human hearts presented a significant increase in total PKCβII and PKCα levels accompanied by their activation. Similarly, we found that of the PKC isozymes expressed in heart, only PKCβII was activated in failing hearts from myocardial infarction- and hypertensive-induced HF rats. Further, we showed that PKCβII isozyme activation directly regulates proteasomal activity and PQC. PKCβII (but not αPKC or PKCβI) activation results in phosphorylation of the purified 20S proteasome *in vitro* with a reduction in its activity (Fig. 2A). The functional consequence of PKCβII-induced 20S proteasome phosphorylation was also demonstrated in isolated neonatal cardiomyocytes since both βIIV5-3, a PKCβII-specific inhibitor (but not selective inhibitors for α, βI or PKCε) and PKCβ knockdown using siRNA abrogated PMA-induced proteasomal dysfunction and the accumulation of damaged proteins (Fig. 2B–C). Since proteasomal activity is regulated by multiple factors, such as intracellular ATP levels [26] and post-translational modification of the proteasome [28], [29], [30], [31], the *in vitro* findings might not reflect proteasomal regulation *in vivo*. Thus, we next examined whether PKCβII activation disrupts cardiac PQC related to UPS dysfunction in myocardial infarction- and hypertensive-induced HF rats. Both HF animal models displayed accumulated misfolded proteins associated with proteasomal dysfunction. Indeed, PKCβII, which is over-activated in these failing rat hearts, co-immunoprecipitated with the 20S proteasome and was found to have decreased activity (Fig. 3B). Taken together, *in vitro* cell culture and *in vivo* data identify PKCβII as a key enzyme in down-regulating proteasomal activity, which resulted in disrupting cardiac PQC and worsening HF with increased mortality. Considering this scenario, the usage of selective inhibitors of PKCβII could provide a new pharmacological tool against PQC disruption in heart failure.

The use of proteasome inhibitors for therapeutic purposes has been proposed based on the major role of proteasomes in degrading intracellular proteins involved in uncontrolled cell proliferation and growth [32]. In cardiac disease, both beneficial and detrimental effects were reported for pharmacologically-induced proteasome inhibition. While most studies on pressure-overload hypertrophy have shown that systemic proteasome inhibition prevented or reversed concentric cardiac hypertrophy with no impact on cardiac function [33], [34], cardiotoxic effects were attributed to proteasome inhibition in normal and ischemic hearts [26], [35], [36]. These findings raise important questions regarding the degree of proteasomal inhibition, which inhibitor should be used (reversible or irreversible) and the appropriate therapeutic time window (when and for how long) such an inhibitor should be used. The latter is of particular interest, since long-term use of proteasome inhibitors seem counterintuitive based on UPS dysfunction- mediated PQC disruption reported in chronic cardiac proteinopathies [37], [38] and as reported here in human HF. Further, the chronic use of the proteasome inhibitor, bortezomib, for chemotherapy was reported to cause cardiac complications ranging from cardiotoxicity to HF in some cancer patients [39]. Relevant to these observations, we found that sustained bortezomib treatment resulted in 100% mortality in myocardial infarction-induced HF rats (fractional shortening below 25%) and blocked PKCβII-related cardioprotective effects. Since control animals treated with bortezomib did not die, these findings highlight the contribution of intact UPS function to cardiac integrity and strengthen our premise that the improvements in heart function/survival induced by PKCβII inhibition is mediated in part by protecting proteasomal function. It is also important to emphasize that most HF patients are elderly and that proteasome activity declines with ageing [40], which might suggest that aged hearts are more susceptible to UPS dysfunction and PQC disruption.

Considering that proteasomal dysfunction is likely responsible for PQC disruption in HF, therapies that prevent or reverse selectively HF-induced proteasomal dysfunction may be of value for these patients. Our data in two models of HF in rats suggest that sustained inhibition of PKCβII may provide such novel treatment for HF, since selective inhibition of PKCβII with βIIV5-3 appears safe, even after many weeks of treatment [9], [11], [17]. We showed in animal models that inhibition of PKCβII with βIIV5-3 treatment re-established cardiac PQC, and not only prevented further deterioration of cardiac function, but actually improved ventricular function (Fig. 3H) and prolonged the life span of two rat models of HF with etiologies most common to HF in humans (Fig. 4C and 5F). These results support a model in which the actions of PKCβII in hypertrophied and failing hearts involve primarily the inactivation of the proteasome. Further studies investigating both direct and indirect proteasomal regulation by PKCβII during heart failure progression are required. However, we cannot exclude the possibility that PKCβII exerts other effects that contribute to this pathology. Also, the contribution of other proteolytic systems such as autophagic/lysosomal pathways to cardiac protein quality control in HF should be considered.

Taken together, our data suggest that PKCβII-mediated impairment of cardiac PQC may be critical, at least in part, for the development of cardiac dysfunction in failing hearts. In addition, re-establishment of proteasomal function and PQC with βIIV5-3 treatment suggests specific PKCβII inhibition may be a valuable therapeutic approach for patients with HF.

## Methods

### Ethics statement

The animal protocols were approved by the Stanford University Institutional Animal Care and Use Committee (Protocol ID: 14746) and by the Ethical Committee of the School of Physical Education and Sport of the University of São Paulo, Brazil (Protocol ID: 2009/13). Human biopsies were taken according to the procedure approved by the Human Ethical Committee in Brazil (Protocol ID: CAPP2409/04/029) and USA (IRB number: 350, protocol ID: 96726). Written informed consent was also obtained from all patients undergoing aortic valve replacement surgery.

### Myocardial infarction-induced heart failure model

Myocardial infarction was induced by ligation of the left anterior descending coronary artery (LAD) in Wistar normotensive rats at 12 weeks of age, as described [41]. After the LAD surgery, animals were followed up to 10 weeks to establish the time window of proteasome activity and protein quality control. In addition, to determine the effect of sustained PKCβII inhibition on PQC during heart failure, another group of animals with fractional shortening <25% (MI-HF) was treated between the ages of 16 and 22 weeks with TAT_47–57_-βIIV5-3 (3 mg/kg/day) or with equimolar concentration of TAT_47–57_-carrier peptide, using Alzet osmotic pumps, which were replaced every two weeks. The sham-infarcted group was subjected to TAT treatment as a negative control. Echocardiography (Acuson Sequoia, 14-MHz) to evaluate fractional shortening was performed 10 weeks after LAD surgery as well as before and after sustained PKCβII inhibition. In addition, MI-HF rats were treated between the ages of 16 and 22 weeks with a specific proteasome inhibitor (bortezomib, 0.2 mg/kg, thrice weekly) either alone or together with βIIV5-3. The bortezomib dose was previously shown to produce blood concentrations of bortezomib comparable with those seen in humans [36], [42]. All the biochemical analyses were performed in the ventricular remote area.

### Hypertension-induced model of heart failure

Male Dahl rats were fed with an 8% NaCl-containing diet (high salt diet) or with a 0.3% NaCl low salt diet from the age of 6 weeks onward, as described [13]. All peptides were delivered using osmotic pumps implanted subcutaneously and replaced every two weeks, Dahl rats were treated between the ages of 11 and 17 weeks with the selective PKCβI inhibitor peptide, TAT_47–57_-βIV5-3 (3 mg/kg/day); the selective PKCβII inhibitor peptide, TAT_47–57_-βIIV5-3 (3 mg/kg/day); the selective PKCε inhibitor peptide, TAT_47–57_-εV1-2 (3 mg/kg/day); or an equimolar concentration of TAT_47–57_ carrier peptide alone (1.6 mg/kg/day), as a control.

### Cell culture

Cardiac myocytes were isolated from 1-day-old Sprague-Dawley rat litters, as described [43]. Short interfering RNA was transfected into cardiac myocytes as described [43].

### Proteasome phosphorylation assay

1 ug of purified 20S proteasome (PW8720, Enzo Lif Sci, PA) was incubated with 50 ng of recombinant PKCα, PKCβI, PKCβII or PKCε (Cell Signaling, MA) in assay buffer (25 mM Tris-HCl, pH 7.5, 1 mM CaCl_2_, 20 mM MgCl_2_, 1 mM DTT, 25 nM ATP) at 37°C for 30 minutes. Proteasome phosphorylation was evaluated using serine/threonine phosphorylation antibody (1∶1000) and [γ^32^P] ATP incorporation, as described [44]. Histone phosphorylation was used to check the effectiveness of different PKC isozymes.

### Kinase assay

The kinase assay was performed as described [44].

### Proteasome activity

ATP-dependent (26S) and -independent (20S) chymotrypsin-like activity of the proteasome was assayed in the total lysate from heart or isolated cardiomyocyte using the fluorogenic peptide Suc-Leu-Leu-Val-Tyr-7-amido-4-methylcoumarin (LLVY-AMC, 25 µM) in a microtiter plate (FlexStation II384, Molecular Device Inc, CA), in Tris-HCl buffer (25 mmol/L, pH 7.5). Kinetic analyses were carried out using 50 µg of protein for 30 min at 37°C in the absence and presence of 25 µmol/L ATP plus 5.0 mmol/L MgCl_2_, with the difference attributed to ATP-dependent proteasomal activity. Excitation/emission wavelengths were 350/440 nm. Data were normalized by proteasomal activity in the presence of 2 µmol/L of epoxomicin (a selective proteasome inhibitor). The purified 20S proteasome activity (Fig. 2A) was carried out after finishing the *in vitro* proteasome phosphorylation assay (see method for proteasome phosphorylation assay).

### Cellular oxidized proteins

Protein oxidation was determined as previously described [45]. We evaluated oxidatively modified proteins using an Oxyblot kit (S7150, Millipore, MA). Samples were normalized by GAPDH and expressed as percent control.

### Statistics

Data are expressed as mean ± s.e.m. One-way analysis of variance (ANOVA) with *post-hoc* testing by Tukey was used to analyze data from Fig. 1b–e,i, 2a–c, 3a,c,e–g, 5b–e and 6a–c,e. Two-way repeated measures analysis of variance (ANOVA) with *post-hoc* testing by Tukey was used to analyze data from Fig. 3h. Student's *t*-test (one-tailed distribution/two-sample equal variance) was used to analyze data from Fig. 1j and 3e. Linear regression analysis and correlation test by Pearson's method were used to assess concordance of Fig. 1f,g and 6d. Survival was analyzed by the standard Kaplan-Meier analysis with log-rank test. A value of *P*<0.05 was considered significant.

### Reagents, peptide synthesis, electron microscopy, tissue fractionation, immunoprecipitation, immunoblot assays and human samples

Detailed methods can be found in the Supporting Information S1.

## Supporting Information

Figure S1 **PKCβII inhibition improved ATP-dependent proteasomal activity and decreased hydrogen peroxide-induced accumulation of oxidized proteins and cell death in cultured neonatal cardiomyocytes.** **a**. Schematic panel of PKCβII inhibition and hydrogen peroxide challenge in cultured neonatal cardiomyocytes. **b**. Cultured neonatal cardiomyocytes were pre-treated with TAT or βIIV5-3 plus/minus epoxomicin and challenged with hydrogen peroxide for 5 min. Proteasomal activity, oxidized proteins accumulation and cell death were evaluated after 24 hrs. Cell death was evaluated by lactate dehydrogenase (LDH) release assay in the medium after 24 hrs. Epoxomicin abrogated the βIIV5-3 cytoprotective effect. Error bars indicate SEM. *, p<0.05 compared to control (non-treated cells). §, p<0.05 compared to βIIV5-3-treated cells. Data were analyzed by one-way analysis of variance (ANOVA) with *post-hoc* testing by Tukey.
(DOC)

Figure S2 **Sustained treatment with βIIV5-3 decreased PKCβII translocation to active fraction in myocardial infarction-induced heart failure rats.** The ratio of translocation of PKC to active fraction (Triton-soluble proteins or the particulate fraction/total fraction) in 22-week old rats (10 wks after MI surgery) (n = 6 per group). Total and Triton-soluble fractions were normalized against GAPDH and Gαo, respectively. Error bars indicate SEM. *, p<0.05 compared to control (sham rats, trace). §, p<0.05 compared to βIIV5-3-treated heart failure rats. Total PKC levels and translocations were analyzed by one-way analysis of variance (ANOVA) with *post-hoc* testing by Tukey.
(DOC)

Figure S3 **Sustained βIIV5-3 treatment decreased α-β-crystallin, HSP27, cleaved caspase-3, p53 and IkB protein levels in myocardial infarction-induced heart failure.** **a**. Representative blots of 20S proteasome subunits (α5/7, β1, β5 and β7), α-β-crystallin, HSP27, caspase-3, cleaved caspase-3, p53, IkB and GAPDH protein levels in heart samples from 22 week-old rats (10 wks after MI surgery) (n = 6 per group). **b**. Cardiac proteasome 20S proteasome subunits (α5/7, β1, β5 and β7), **c**. α-β-crystallin, **d**. HSP27, **e**. HSP 90, **f**. Ratio of cardiac cleaved caspase-3/caspase-3, **g**. p53 and **h**. IkB were measured in left ventricle tissue from 22 week-old myocardial infarction-induced heart failure (10 wks after MI surgery) TAT-treated (gray bar), βIIV5-3-treated (green bar) and control (sham, white bar) rats. Data were normalized against GAPDH. Error bars indicate SEM. *, p<0.05 compared to control (sham). §, p<0.05 compared to βIIV5-3-treated heart failure rats. Data were analyzed by one-way analysis of variance (ANOVA) with *post-hoc* testing by Tukey.
(DOC)

Table S1 **Individual characteristics of left ventricular remodeling and heart failure patients.**
(DOC)

Supporting Information S1 Reagents, peptide synthesis, electron microscopy, tissue fractionation, immunoprecipitation, immunoblot assays and human samples.
(DOC)
