# Supplementary material for: Protein Quality Control Disruption by PKCβII in Heart Failure; Rescue by the Selective PKCβII Inhibitor, βIIV5-3
Source: PLoS One. 2012 Mar 30;7(3):e33175. doi: 10.1371/journal.pone.0033175 (PMC3316563; doi:10.1371/journal.pone.0033175)
Supplement: Figure S1 — PKCβII inhibition improved ATP-dependent proteasomal activity and decreased hydrogen peroxide-induced accumulation of oxidized proteins and cell death in cultured neonatal cardiomyocytes. a. Schematic panel of PKCβII inhibition and hydrogen peroxide challenge in cultured neonatal cardiomyocytes. b. Cultured neonatal cardiomyocytes were pre-treated with TAT or βIIV5-3 plus/minus epoxomicin and challenged with hydrogen peroxide for 5 min. Proteasomal activity, oxidized proteins accumulation and cell death were evaluated after 24 hrs. Cell death was evaluated by lactate dehydrogenase (LDH) release assay in the medium after 24 hrs. Epoxomicin abrogated the βIIV5-3 cytoprotective effect. Error bars indicate SEM. *, p<0.05 compared to control (non-treated cells). §, p<0.05 compared to βIIV5-3-treated cells. Data were analyzed by one-way analysis of variance (ANOVA) with post-hoc testing by Tukey. (DOC) [file pone.0033175.s001.doc]

Supporting Figure S1


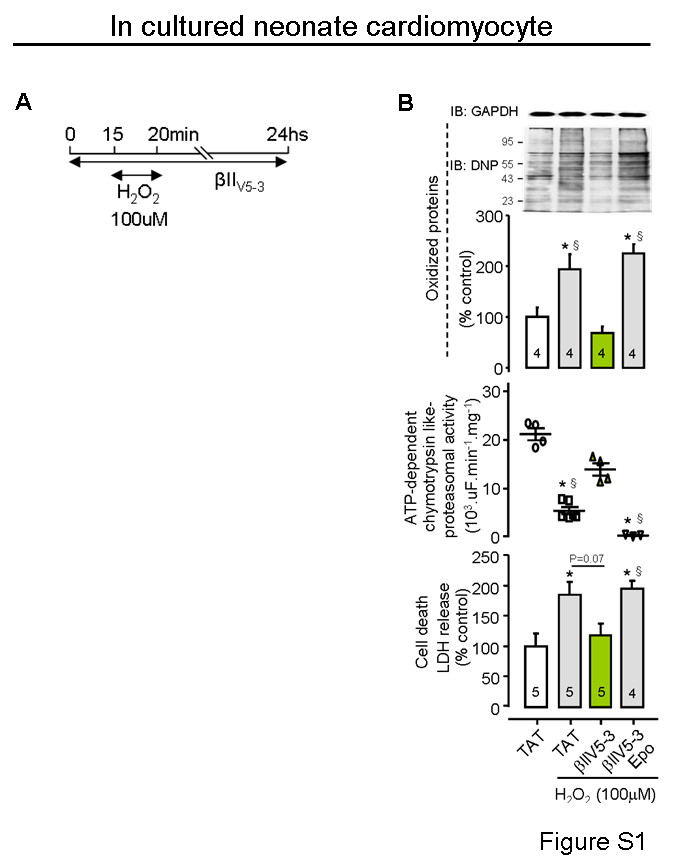


**Figure S1: PKCβII inhibition improved ATP-dependent proteasomal activity and decreased hydrogen peroxide-induced accumulation of oxidized proteins and cell death in cultured neonatal cardiomyocytes.** **a.** Schematic panel of PKCβII inhibition and hydrogen peroxide challenge in cultured neonatal cardiomyocytes. **b.** Cultured neonatal cardiomyocytes were pre-treated with TAT or βIIV5-3 plus/minus epoxomicin and challenged with hydrogen peroxide for 5 min. Proteasomal activity, oxidized proteins accumulation and cell death were evaluated after 24 hrs. Cell death was evaluated by lactate dehydrogenase (LDH) release assay in the medium after 24 hrs. Epoxomicin abrogated the βIIV5-3 cytoprotective effect. Error bars indicate SEM. *, p<0.05 compared to control (non-treated cells). §, p<0.05 compared to βIIV5-3-treated cells. Data were analyzed by one-way analysis of variance (ANOVA) with *post-hoc* testing by Tukey.
