# Supplementary material for: Protein Quality Control Disruption by PKCβII in Heart Failure; Rescue by the Selective PKCβII Inhibitor, βIIV5-3
Source: PLoS One. 2012 Mar 30;7(3):e33175. doi: 10.1371/journal.pone.0033175 (PMC3316563; doi:10.1371/journal.pone.0033175)
Supplement: Figure S2 — Sustained treatment with βIIV5-3 decreased PKCβII translocation to active fraction in myocardial infarction-induced heart failure rats. The ratio of translocation of PKC to active fraction (Triton-soluble proteins or the particulate fraction/total fraction) in 22-week old rats (10 wks after MI surgery) (n = 6 per group). Total and Triton-soluble fractions were normalized against GAPDH and Gαo, respectively. Error bars indicate SEM. *, p<0.05 compared to control (sham rats, trace). §, p<0.05 compared to βIIV5-3-treated heart failure rats. Total PKC levels and translocations were analyzed by one-way analysis of variance (ANOVA) with post-hoc testing by Tukey. (DOC) [file pone.0033175.s002.doc]

Supporting Information S2


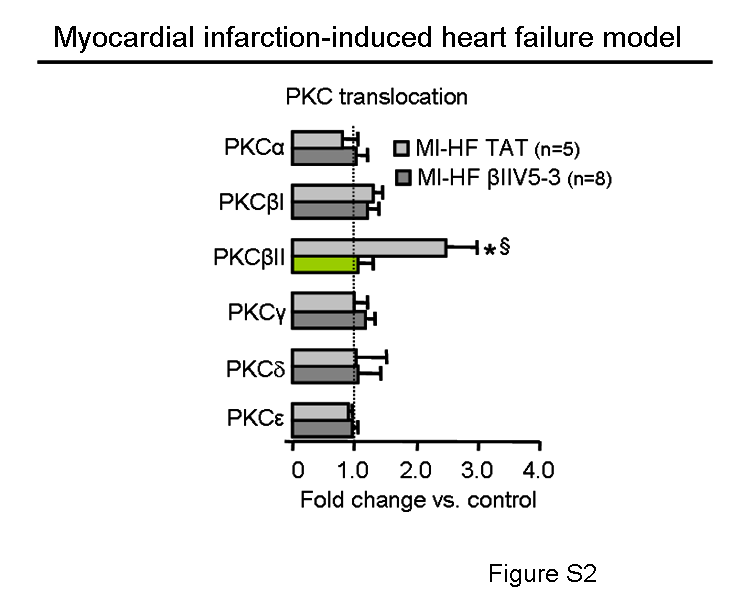


**Figure S2: Sustained treatment with βIIV5-3 decreased PKCβII translocation to active fraction in myocardial infarction-induced heart failure rats.** The ratio of translocation of PKC to active fraction (Triton-soluble proteins or the particulate fraction/total fraction) in 22-week old rats (10wks after MI surgery) (n=6 per group). Total and Triton-soluble fractions were normalized against GAPDH and Gαo, respectively. Error bars indicate SEM. *, p<0.05 compared to control (sham rats, trace). §, p<0.05 compared to βIIV5-3**-**treated heart failure rats. Total PKC levels and translocations were analyzed by one-way analysis of variance (ANOVA) with *post-hoc* testing by Tukey.
