# Supplementary material for: Protein Quality Control Disruption by PKCβII in Heart Failure; Rescue by the Selective PKCβII Inhibitor, βIIV5-3
Source: PLoS One. 2012 Mar 30;7(3):e33175. doi: 10.1371/journal.pone.0033175 (PMC3316563; doi:10.1371/journal.pone.0033175)
Supplement: Figure S3 — Sustained βIIV5-3 treatment decreased α-β-crystallin, HSP27, cleaved caspase-3, p53 and IkB protein levels in myocardial infarction-induced heart failure. a. Representative blots of 20S proteasome subunits (α5/7, β1, β5 and β7), α-β-crystallin, HSP27, caspase-3, cleaved caspase-3, p53, IkB and GAPDH protein levels in heart samples from 22 week-old rats (10 wks after MI surgery) (n = 6 per group). b. Cardiac proteasome 20S proteasome subunits (α5/7, β1, β5 and β7), c. α-β-crystallin, d. HSP27, e. HSP 90, f. Ratio of cardiac cleaved caspase-3/caspase-3, g. p53 and h. IkB were measured in left ventricle tissue from 22 week-old myocardial infarction-induced heart failure (10 wks after MI surgery) TAT-treated (gray bar), βIIV5-3-treated (green bar) and control (sham, white bar) rats. Data were normalized against GAPDH. Error bars indicate SEM. *, p<0.05 compared to control (sham). §, p<0.05 compared to βIIV5-3-treated heart failure rats. Data were analyzed by one-way analysis of variance (ANOVA) with post-hoc testing by Tukey. (DOC) [file pone.0033175.s003.doc]

Supporting Figure S3


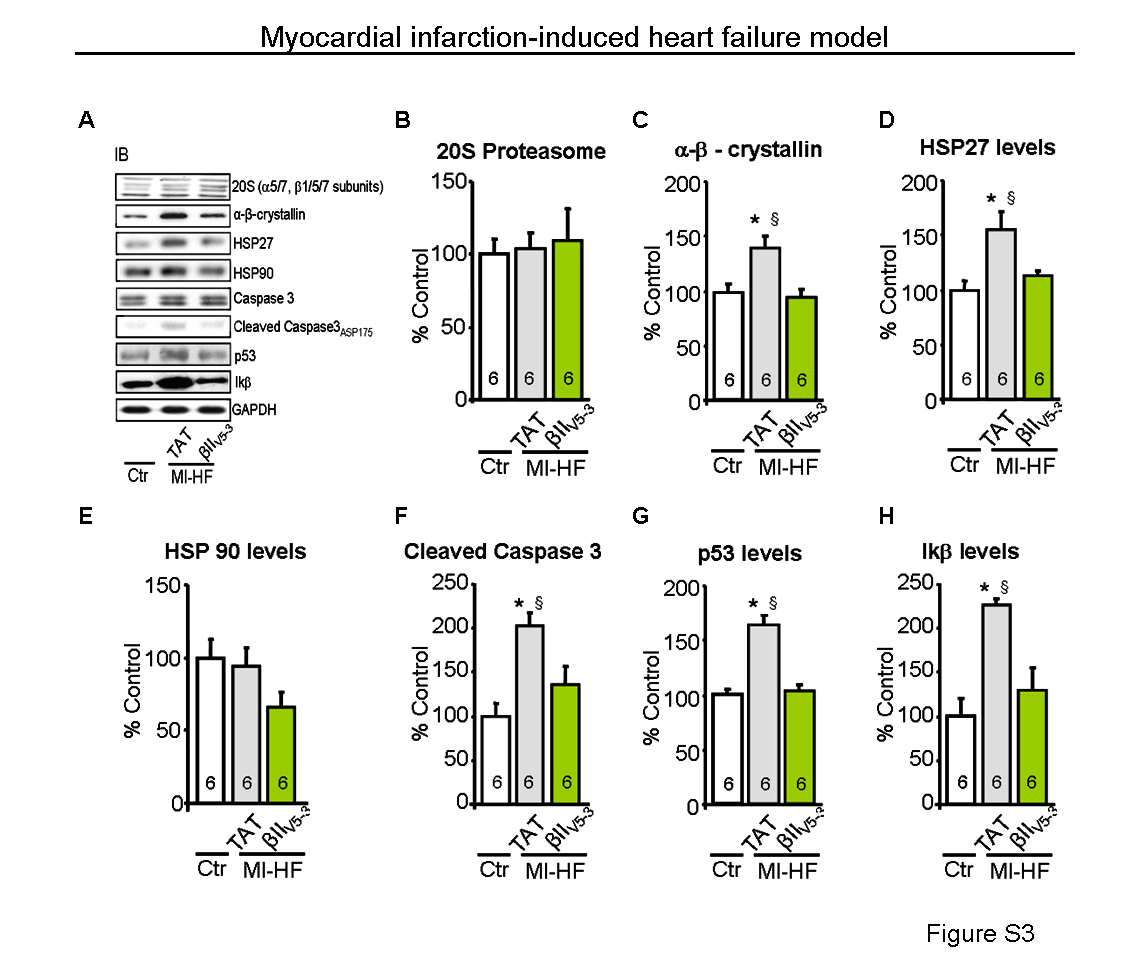


**Figure S3: Sustained βIIV5-3 treatment decreased α-β-crystallin, HSP27, cleaved caspase-3, p53 and Ikβ protein levels in myocardial infarction-induced heart failure. a.** Representative blots of 20S proteasome subunits (α5/7, β1, β5 and β7), α-β-crystallin, HSP27, caspase-3, cleaved caspase-3, p53, Ikβ and GAPDH protein levels in heart samples from 22 week-old rats (10wks after MI surgery) (n=6 per group). **b.** Cardiac proteasome 20S proteasome subunits (α5/7, β1, β5 and β7), **c.** α-β-crystallin, **d.** HSP27, **e.** HSP90, **f.** Ratio of cardiac cleaved caspase-3/caspase-3, **g.** p53 and **h.** Ikβ were measured in left ventricle tissue from 22 week-old myocardial infarction-induced heart failure (10wks after MI surgery) TAT-treated (gray bar), βIIV5-3-treated (green bar) and control (sham, white bar) rats. Data were normalized against GAPDH. Error bars indicate SEM. *, p<0.05 compared to control (sham). §, p<0.05 compared to βIIV5-3-treated heart failure rats. Data were analyzed by one-way analysis of variance (ANOVA) with *post-hoc* testing by Tukey.
