# Supplementary figures and images for: Protein Quality Control Disruption by PKCβII in Heart Failure; Rescue by the Selective PKCβII Inhibitor, βIIV5-3
Source: PLoS One. 2012 Mar 30;7(3):e33175. doi: 10.1371/journal.pone.0033175 (PMC3316563; doi:10.1371/journal.pone.0033175)

Supporting Table S4


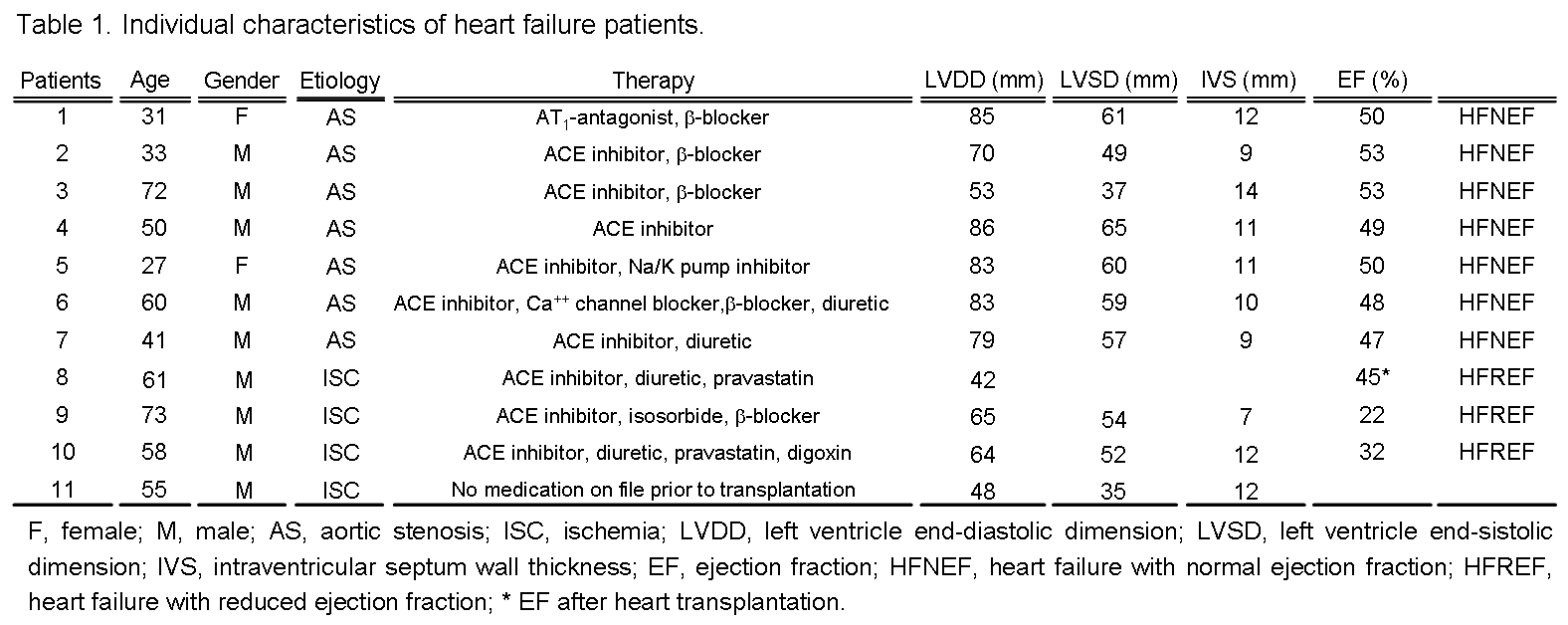

Supplement: Table S1 — Individual characteristics of left ventricular remodeling and heart failure patients. (DOC) [file pone.0033175.s004.doc]
