# Supplementary material for: Protein Quality Control Disruption by PKCβII in Heart Failure; Rescue by the Selective PKCβII Inhibitor, βIIV5-3
Source: PLoS One. 2012 Mar 30;7(3):e33175. doi: 10.1371/journal.pone.0033175 (PMC3316563; doi:10.1371/journal.pone.0033175)
Supplement: Supporting Information S1 — Reagents, peptide synthesis, electron microscopy, tissue fractionation, immunoprecipitation, immunoblot assays and human samples. (DOC) [file pone.0033175.s005.doc]

Supporting information S5

**Supplementary Methods**

**Reagents.** Proteasomal fluorogenic substrate Suc-Leu-Leu-Val-Tyr-7-amido-4-methylcoumarin (LLVY-AMC, cat. P802), proteasomal inhibitor epoxomicin (cat. PI127), human purified 20S proteasome subunit (PW8720) and anti-20S proteasome core subunits (PW8155) were all purchased from Enzo Lif Sci, PA. Phosphatase and protease inhibitor cocktails were purchased from Sigma-Aldrich. The cardiomyocyte isolation kit was purchased from Cellutron. Antibodies against PKCα (cat. sc-208), PKCβI (cat. sc-209), PKCβII (cat. sc-210), PKCδ (cat. sc-213), PKCγ (cat. sc-211), PKCε (cat. sc-214), HSP27 (cat. sc-1048), ubiquitinated proteins (cat. sc-8017) and GAPDH (cat. sc-137179) were from Santa Cruz Biotechnology, CA. Anti-soluble oligomer A11 (cat. AB9234) was from Millipore, MA. Caspase 3 (cat. 9662), anti-cleaved caspase 3 (cat. 9664), Ikβ (cat. 9242), p53 (cat. 2524) and anti-serine and threonine phosphorylation antibodies were from Cell Signaling Technology, MA. Recombinant PKCs were purchased from Cell Signaling Technology, MA. Oxyblot kit detection was from Millipore (cat. S7150). Anti-mouse IgG, anti-rabbit IgG and peroxidase-linked species-specific antibodies were purchased from GE Heathcare, NJ. PKCβ and PKCα siRNA were purchased from Applied Biosystem and Lipofectamine 2000 from Invitrogen. Bortezomib was from LC laboratories, MA (cat. B-1408).

**Peptide synthesis.** αV5-3 (an αPKC-specific inhibitor, corresponding to amino acids 642-647 [QLVIAN]), βIV5-3 (a βIPKC-specific inhibitor, amino acids 646-651 [KLFIMN]), βIIV5-3 (a βIIPKC-specific inhibitor, amino acids 645-650 [QEVIRN]) [1] and εV1-2 (an εPKC-specific inhibitor, amino acids 14-21 [EAVSLKPT]) [2] were synthesized and conjugated to TAT_47-57_ carrier peptide (amino acids 47–57 [YGRKKRRQRRR]) *via* a disulfide bond between Cys residues at the N-terminus of each peptide [3] by American Peptide, Inc. (Sunnyvale, CA). The ability of TAT_47-57_ to deliver the peptides into all the cardiac cells, the efficacy and selectivity of these peptides for the corresponding PKC isozymes have been documented elsewhere [1,4,5,6,7,8,9,10].

**Electron microscopy.** For TEM analysis, TAT- and βIIV5-3-treated rats were sacrificed and hearts perfused with phosphate buffered saline. Small blocks (5 blocks per animal) from a cardiac remote area were cut and fixed with 2% glutaraldehyde and 4% paraformaldehyde in sodium cacodylate buffer, pH 7.3 for 1 hour at room temperature and cut into ~1mm^3^ blocks. After several buffer washes, the samples were post-fixed in 2% osmium tetroxide and 1% uranyl acetate for 2 h, rinsed in water, dehydrated through ascending concentrations of ethanol followed by 100% acetone, and then infiltrated and embedded in Eponate 12. We performed qualitative analysis in at least 10 fields per rat (3 rats per group).

**Tissue fractionation.** Frozen heart tissues were thawed on ice in homogenization buffer (20 mM Tris-HCl [pH 7.5], 2 mM EDTA, 10 mM EGTA, 250 mM sucrose, and protease and phosphatase inhibitors). The homogenates were centrifuged at 100,000 g for 30 min (4°C) to separate the cytoplasmic soluble fraction from the membranous fraction. After the soluble fractions were isolated, the pellets were dissolved in homogenization buffer containing 1% Triton X-100, incubated on ice for 30 min, and subsequently centrifuged at 100,000 g for 30 min (4°C). The Triton-soluble particulate fraction was isolated, and the Triton-insoluble fraction pellets were dissolved in homogenization buffer.

**Immunoprecipitation**. Total lysate of rat heart (500 μg protein) was incubated with the indicated antibodies for 3 h at 4°C, followed by incubation with protein A/G agarose beads (Santa Cruz Biotechnology) for 1 h at 4°C. The immunoprecipitates were separated on SDS–PAGE and transferred onto nitrocellulose membranes. The membranes were then probed with the indicated antibodies.

**Immunoblot.** Immunoblots from total lysate of rat heart or cultured cell homogenates were performed according to Towbin et al. [11]. Samples were loaded and subjected to SDS-PAGE in polyacrylamide gels. After electrophoresis, proteins were electro-transferred to nitrocellulose membrane (Amersham Biosciences, NJ,). Equal loading of samples (25 μg) and even transfer efficiency were monitored with the use of 0.5% Ponceau S staining of the blot membrane. The blot membrane was then incubated in a blocking buffer (5% bovine serum albumin, 10mM Tris-HCl, pH 7.6, 150 mM NaCl, and 0.1% Tween 20) for 2 h at room temperature and then incubated with a specific antibody overnight at 4^o^C overnight. Binding of the primary antibody was detected with the use of peroxidase-conjugated secondary antibodies. The enhanced chemiluminescence reagents (Amersham Biosciences, NJ) were used to visualize the autoradiogram, which was later exposed to photographic film. Quantification analysis of blots was performed with the use of Image J software (Image J Corporation based on NIH image). The protein contents were quantified by the Bradford method [12].

**Cellular soluble oligomers of misfolded proteins.** To assess the levels of soluble oligomers of misfolded proteins, heart tissue was homogenized in lysis buffer and 25μg of total protein was slot-blotted onto PVDF membrane (Immol/Lobilon-P, Millipore, MA) using a Minifold II slot blot apparatus. The membranes were washed 3X with T-TBS (0.05% v/v Tween 20, 10 mmol/L Tris, pH 7.5, 100 mmol/L NaCl) and then blocked for 1 hour (T-TBS, 5% milk). After blocking, blots were incubated for 4 hours with anti-soluble oligomer A11 antibody, an antibody shown to recognize cellular soluble oligomers of misfolded proteins [13]. Blots were then incubated with a secondary anti-IgG rabbit antibody linked to horseradish peroxidase for 2 hours. Protein slots were visualized and quantified as in Western blot. Samples were normalized by ponceau staining and expressed as percent control. This antibody does not recognize monomers of mature fibers of proteins or peptides.

**Human samples.** Small biopsy specimens of left ventricle were obtained from seven patients with aortic stenosis-induced left ventricular remodeling submitted to aortic valve replacement surgery, from four patients with ischemic cardiomyopathy-induced HF and autopsy specimens from 13 non-failing human hearts as controls. Despite preserved systolic function, all patients with aortic stenosis displayed heart failure signs and symptoms, presenting functional class III-IV of New York Heart Association [14]. The autopsy specimens from non-failing human hearts were taken in a time-window of 30 minutes after death. Biopsies were taken according to the procedure approved by the Human Ethical Committee in Brazil (CAPP2409/04/029) and USA (IRB number: 350, protocol ID: 96726). Written Informed consent was also obtained from all patients undergoing aortic valve replacement surgery.
